# 3,3,4,4-Tetra­fluoro-1-[2-(3,3,4,4-tetra­fluoro­pyrrolidin-1-yl)phen­yl]pyrrolidine

**DOI:** 10.1107/S1600536811033757

**Published:** 2011-08-27

**Authors:** Jin Wang, Jun-Wen Zhong, Pei-Lian Liu, Wan-Wan Cao, Zhuo Zeng

**Affiliations:** a School of Chemistry and Environment, South China Normal University, Guangzhou 510006, People’s Republic of China; b Key Laboratory of Organofluorine Chemistry, Shanghai Institute of Organic Chemistry, Chinese Academy of Sciences, Shanghai 200032, People’s Republic of China

## Abstract

The asymmetric unit of the title compound, C_14_H_12_F_8_N_2_, contains one tetra­fluoro­pyrrolidine system and one half-mol­ecule of benzene; the latter, together with a second heterocyclic unit, are completed by symmetry, with a twofold crystallographic axis crossing through both the middle of the bond between the C atoms bearing the heterocyclic rings and the opposite C—C bonds of the whole benzene mol­ecule. The pyrrolidine ring shows an envelope conformation with the apex at the N atom. The dihedral angle between the least-squares plane of this ring and the benzene ring is 36.9 (5)°. There are intra­molecular C—H⋯N inter­actions generating *S*(6) ring motifs. In the crystal structure, the mol­ecules are linked by C—H⋯F inter­actions, forming chains parallel to [010].

## Related literature

For background to the properties of fluorinated and alkyl-fluorinated heterocyclic compounds, see: Babudri *et al.* (2007 ▶). For applications of compounds with fluorinated rings, see: Brambilla (2001 ▶); Hagan (2008 ▶). For the synthesis of related compounds, see: Zeng & Shreeve (2009 ▶). For a description of hydrogen-bonding motifs, see: Bernstein *et al.* (1995 ▶).
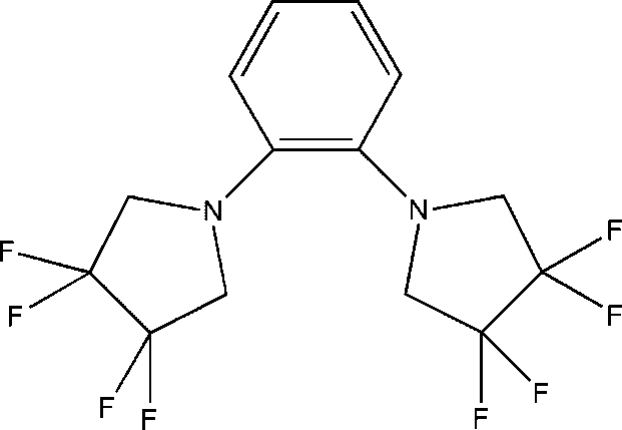


## Experimental

### 

#### Crystal data

C_14_H_12_F_8_N_2_
*M* *_r_* = 360.26
Orthorhombic, 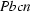

*a* = 8.678 (3) Å
*b* = 9.818 (3) Å
*c* = 17.088 (5) Å
*V* = 1455.9 (8) Å^3^
*Z* = 4
Mo *K*α radiation
μ = 0.17 mm^−1^
*T* = 293 K
0.44 × 0.37 × 0.22 mm

#### Data collection

Bruker SMART CCD area-detector diffractometer
Absorption correction: multi-scan (*SADABS*; Sheldrick, 1996 ▶) *T*_min_ = 0.928, *T*_max_ = 0.963
6762 measured reflections
1283 independent reflections
878 reflections with *I* > 2σ(*I*)
*R*_int_ = 0.036

#### Refinement

*R*[*F* ^2^ > 2σ(*F* ^2^)] = 0.071
*wR*(*F* ^2^) = 0.129
*S* = 0.92
1283 reflections
109 parameters
H-atom parameters constrained
Δρ_max_ = 0.50 e Å^−3^
Δρ_min_ = −0.41 e Å^−3^

### 

Data collection: *SMART* (Bruker, 1997 ▶); cell refinement: *SAINT* (Bruker, 1997 ▶); data reduction: *SAINT*; program(s) used to solve structure: *SHELXS97* (Sheldrick, 2008 ▶); program(s) used to refine structure: *SHELXL97* (Sheldrick, 2008 ▶); molecular graphics: *SHELXTL* (Sheldrick, 2008 ▶); software used to prepare material for publication: *SHELXTL*.

## Supplementary Material

Crystal structure: contains datablock(s) global, I. DOI: 10.1107/S1600536811033757/lr2025sup1.cif

Structure factors: contains datablock(s) I. DOI: 10.1107/S1600536811033757/lr2025Isup2.hkl

Additional supplementary materials:  crystallographic information; 3D view; checkCIF report

## Figures and Tables

**Table 1:** Hydrogen-bond geometry (Å, °)

*D*—H⋯*A*	*D*—H	H⋯*A*	*D*⋯*A*	*D*—H⋯*A*
C1—H1⋯F3^i^	0.93	2.54	3.398 (4)	154
C7—H7*B*⋯N1^ii^	0.97	2.45	3.020 (4)	117

Symmetry codes: (i) 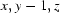; (ii) 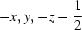.

**Fig. 1.**
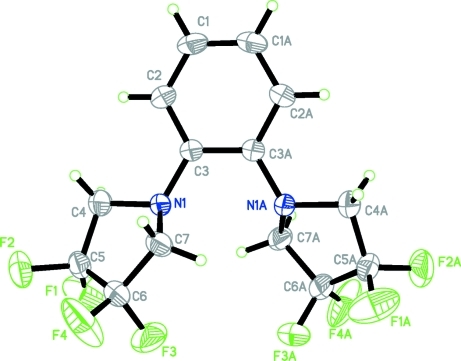
An ORTEP view of the title compound. Ellipsoids are drawn at the 50% probability level.

**Fig. 2.**
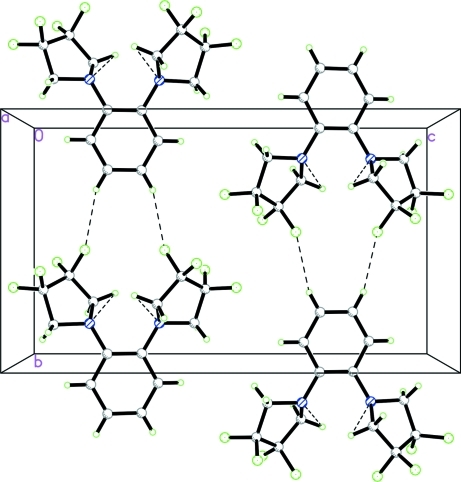
Perspective view of the crystal packing.

## supplementary crystallographic information

### Comment

It is well known that the introduction of a fluorine atom or a fluoroalkyl group into heterocyclic compounds has a profound influence on their chemical, physical and biological properties (Hagan, 2008). Fluorinated ring play an important role in the pharmaceutical and advanced materials fields (Babudri *et al.*, 2007). Many forms of systemic or topical fluoride have been studied and tested for clinical application (Brambilla, 2001). As a part of our studies in this area, we now report the synthesis and structure of the title compound.

An *ORTEP* view of the title compound, C_14_H_12_F_8_N_2_, is depicted in Fig. 1. The asymmetric unit contains one tetrafluoropyrrolidin system and a half-molecule of benzene. The whole molecule is generated by rotation around a 2-fold crystallographic axis crossing through, both, the middle of the bond between the carbon atoms bearing the heterocyclic rings (C3 and C3a) and the opposite C1—C1a bond. The pyrrolidine ring shows an envelope conformation with the apex at the N1 atom, the dihedral angle between the least-squares plane of this ring and the benzene moiety is 36.9 (5)°.

Intramolecular C7—H7B···N1 interactions generating S(6) ring motifs are present (Table 1). In the crystal the molecules are linked by C1—H1···F3 interactions (symmetry operation x, y - 1, z) forming chains parallel to [010] direction (Table 1 and Fig. 2).

### Experimental

The compound was prepared using a slightly variation of our previously reported procedure (Zeng & Shreeve, 2009). Single crystals of the title compound were obtained by slow evaporation from dichloromethane at room temperature.

### Refinement

H atoms were placed in calculated positions C—H = 0.93 or 0.97 Å and refined as riding with *U*_iso_(H) = 1.2 *U*_iso_(C). The 200 reflection with Δ F^2^/ e.s.d. = 14.8 has been omitted from the refinement.

### Crystal data

**Table:**

C_14_H_12_F_8_N_2_	*F*(000) = 728
*M**_r_* = 360.26	*D*_x_ = 1.644 Mg m^−^^3^
Orthorhombic, *P**b**c**n*	Mo *K*α radiation, λ = 0.71073 Å
Hall symbol: -P 2n 2ab	Cell parameters from 1256 reflections
*a* = 8.678 (3) Å	θ = 2.4–20.4°
*b* = 9.818 (3) Å	µ = 0.17 mm^−^^1^
*c* = 17.088 (5) Å	*T* = 293 K
*V* = 1455.9 (8) Å^3^	Block, colourless
*Z* = 4	0.44 × 0.37 × 0.22 mm

### Data collection

**Table:**

Bruker SMART CCD area-detector diffractometer	1283 independent reflections
Radiation source: fine-focus sealed tube	878 reflections with *I* > 2σ(*I*)
graphite	*R*_int_ = 0.036
φ and ω scans	θ_max_ = 25.0°, θ_min_ = 2.4°
Absorption correction: multi-scan (*SADABS*; Sheldrick, 1996)	*h* = −8→10
*T*_min_ = 0.928, *T*_max_ = 0.963	*k* = −10→11
6762 measured reflections	*l* = −20→16

### Refinement

**Table:**

Refinement on *F*^2^	Primary atom site location: structure-invariant direct methods
Least-squares matrix: full	Secondary atom site location: difference Fourier map
*R*[*F*^2^ > 2σ(*F*^2^)] = 0.071	Hydrogen site location: inferred from neighbouring sites
*wR*(*F*^2^) = 0.129	H-atom parameters constrained
*S* = 0.92	*w* = 1/[σ^2^(*F*_o_^2^) + (0.005*P*)^2^ + 3.940*P*] where *P* = (*F*_o_^2^ + 2*F*_c_^2^)/3
1283 reflections	(Δ/σ)_max_ = 0.022
109 parameters	Δρ_max_ = 0.50 e Å^−^^3^
0 restraints	Δρ_min_ = −0.41 e Å^−^^3^

### Special details

**Table:**

Geometry. All e.s.d.'s (except the e.s.d. in the dihedral angle between two l.s. planes) are estimated using the full covariance matrix. The cell e.s.d.'s are taken into account individually in the estimation of e.s.d.'s in distances, angles and torsion angles; correlations between e.s.d.'s in cell parameters are only used when they are defined by crystal symmetry. An approximate (isotropic) treatment of cell e.s.d.'s is used for estimating e.s.d.'s involving l.s. planes.
Refinement. Refinement of *F*^2^ against ALL reflections. The weighted *R*-factor *wR* and goodness of fit *S* are based on *F*^2^, conventional *R*-factors *R* are based on *F*, with *F* set to zero for negative *F*^2^. The threshold expression of *F*^2^ > σ(*F*^2^) is used only for calculating *R*-factors(gt) *etc*. and is not relevant to the choice of reflections for refinement. *R*-factors based on *F*^2^ are statistically about twice as large as those based on *F*, and *R*-factors based on ALL data will be even larger.

### Fractional atomic coordinates and isotropic or equivalent isotropic displacement parameters (Å^2^)

**Table:**

	*x*	*y*	*z*	*U*_iso_*/*U*_eq_	
F1	−0.1645 (4)	−0.1228 (3)	−0.4379 (2)	0.1363 (14)	
F2	0.0115 (4)	−0.1911 (3)	−0.51367 (13)	0.1146 (11)	
F3	0.0249 (6)	−0.0376 (3)	−0.34538 (19)	0.181 (2)	
F4	0.2023 (4)	−0.0915 (4)	−0.42295 (19)	0.1626 (19)	
N1	−0.0183 (3)	−0.3376 (3)	−0.33165 (14)	0.0491 (7)	
C1	−0.0181 (5)	−0.7079 (3)	−0.2889 (2)	0.0673 (11)	
H1	−0.0338	−0.7897	−0.3151	0.081*	
C2	−0.0311 (4)	−0.5864 (3)	−0.3283 (2)	0.0586 (10)	
H2	−0.0529	−0.5872	−0.3815	0.070*	
C3	−0.0126 (4)	−0.4627 (3)	−0.29036 (18)	0.0472 (8)	
C4	−0.0670 (5)	−0.3406 (4)	−0.4133 (2)	0.0697 (12)	
H4A	−0.1751	−0.3646	−0.4179	0.084*	
H4B	−0.0060	−0.4047	−0.4434	0.084*	
C5	−0.0388 (5)	−0.1976 (4)	−0.4393 (2)	0.0663 (11)	
C6	0.0846 (6)	−0.1425 (4)	−0.3851 (2)	0.0743 (12)	
C7	0.1226 (5)	−0.2548 (4)	−0.3301 (2)	0.0636 (10)	
H7A	0.2110	−0.3065	−0.3481	0.076*	
H7B	0.1432	−0.2204	−0.2779	0.076*	

### Atomic displacement parameters (Å^2^)

**Table:**

	*U*^11^	*U*^22^	*U*^33^	*U*^12^	*U*^13^	*U*^23^
F1	0.089 (2)	0.106 (2)	0.214 (4)	0.0321 (19)	0.015 (2)	0.049 (2)
F2	0.171 (3)	0.124 (2)	0.0491 (14)	−0.027 (2)	0.0102 (17)	0.0096 (14)
F3	0.380 (7)	0.0575 (16)	0.106 (2)	0.053 (3)	−0.059 (3)	−0.0167 (16)
F4	0.153 (3)	0.208 (4)	0.127 (3)	−0.099 (3)	−0.046 (2)	0.102 (3)
N1	0.0642 (19)	0.0434 (14)	0.0395 (14)	−0.0035 (14)	−0.0065 (14)	0.0017 (12)
C1	0.075 (3)	0.0410 (18)	0.086 (3)	−0.005 (2)	0.006 (2)	−0.0105 (18)
C2	0.069 (3)	0.0468 (19)	0.060 (2)	−0.0026 (18)	−0.0019 (19)	−0.0090 (17)
C3	0.052 (2)	0.0416 (17)	0.0478 (17)	−0.0006 (16)	−0.0009 (16)	−0.0011 (14)
C4	0.097 (3)	0.063 (2)	0.049 (2)	−0.005 (2)	−0.020 (2)	0.0015 (19)
C5	0.084 (3)	0.069 (2)	0.047 (2)	0.009 (2)	0.003 (2)	0.0102 (18)
C6	0.107 (4)	0.058 (2)	0.058 (2)	−0.016 (2)	−0.002 (2)	0.011 (2)
C7	0.078 (3)	0.053 (2)	0.060 (2)	−0.012 (2)	−0.009 (2)	0.0106 (18)

### Geometric parameters (Å, °)

**Table:**

F1—C5	1.315 (5)	C2—C3	1.385 (4)
F2—C5	1.344 (4)	C2—H2	0.9300
F3—C6	1.337 (5)	C3—C3^i^	1.397 (6)
F4—C6	1.309 (5)	C4—C5	1.493 (5)
N1—C3	1.418 (4)	C4—H4A	0.9700
N1—C4	1.458 (4)	C4—H4B	0.9700
N1—C7	1.468 (5)	C5—C6	1.516 (6)
C1—C1^i^	1.364 (7)	C6—C7	1.485 (5)
C1—C2	1.374 (5)	C7—H7A	0.9700
C1—H1	0.9300	C7—H7B	0.9700
			
C3—N1—C4	117.9 (3)	F1—C5—F2	105.1 (3)
C3—N1—C7	116.2 (3)	F1—C5—C4	112.5 (4)
C4—N1—C7	105.7 (3)	F2—C5—C4	112.3 (3)
C1^i^—C1—C2	119.7 (2)	F1—C5—C6	112.0 (4)
C1^i^—C1—H1	120.1	F2—C5—C6	109.4 (4)
C2—C1—H1	120.1	C4—C5—C6	105.6 (3)
C1—C2—C3	121.5 (3)	F4—C6—F3	105.0 (4)
C1—C2—H2	119.3	F4—C6—C7	115.0 (4)
C3—C2—H2	119.3	F3—C6—C7	109.7 (3)
C2—C3—C3^i^	118.7 (2)	F4—C6—C5	112.6 (3)
C2—C3—N1	121.5 (3)	F3—C6—C5	108.2 (4)
C3^i^—C3—N1	119.80 (16)	C7—C6—C5	106.2 (3)
N1—C4—C5	102.6 (3)	N1—C7—C6	102.4 (3)
N1—C4—H4A	111.2	N1—C7—H7A	111.3
C5—C4—H4A	111.2	C6—C7—H7A	111.3
N1—C4—H4B	111.2	N1—C7—H7B	111.3
C5—C4—H4B	111.2	C6—C7—H7B	111.3
H4A—C4—H4B	109.2	H7A—C7—H7B	109.2
			
C1^i^—C1—C2—C3	−1.8 (8)	F2—C5—C6—F4	5.7 (5)
C1—C2—C3—C3^i^	−3.0 (7)	C4—C5—C6—F4	126.7 (4)
C1—C2—C3—N1	176.7 (4)	F1—C5—C6—F3	5.1 (5)
C4—N1—C3—C2	9.0 (5)	F2—C5—C6—F3	121.2 (4)
C7—N1—C3—C2	−118.0 (4)	C4—C5—C6—F3	−117.7 (4)
C4—N1—C3—C3^i^	−171.2 (4)	F1—C5—C6—C7	122.8 (4)
C7—N1—C3—C3^i^	61.8 (5)	F2—C5—C6—C7	−121.1 (4)
C3—N1—C4—C5	−172.8 (3)	C4—C5—C6—C7	0.0 (5)
C7—N1—C4—C5	−40.9 (4)	C3—N1—C7—C6	173.7 (3)
N1—C4—C5—F1	−98.1 (4)	C4—N1—C7—C6	40.8 (4)
N1—C4—C5—F2	143.5 (4)	F4—C6—C7—N1	−149.4 (4)
N1—C4—C5—C6	24.4 (4)	F3—C6—C7—N1	92.5 (4)
F1—C5—C6—F4	−110.5 (5)	C5—C6—C7—N1	−24.1 (4)

Symmetry codes: (i) −*x*, *y*, −*z*−1/2.

### Hydrogen-bond geometry (Å, °)

**Table:**

*D*—H···*A*	*D*—H	H···*A*	*D*···*A*	*D*—H···*A*
C1—H1···F3^ii^	0.93	2.54	3.398 (4)	154.
C7—H7B···N1^i^	0.97	2.45	3.020 (4)	117.

Symmetry codes: (ii) *x*, *y*−1, *z*; (i) −*x*, *y*, −*z*−1/2.

## References

[bb1] Babudri, F., Farinola, G. M., Naso, F. & Ragni, R. (2007). *Chem. Commun.* pp. 1003–1022. 10.1039/b611336b 17325792

[bb2] Bernstein, J., Davis, R. E., Shimoni, L. & Chang, N.-L. (1995). *Angew. Chem. Int. Ed. Engl.* **34**, 1555–1573.

[bb3] Brambilla, E. (2001). *Caries Res.* **35**, 6–9. 10.1159/000049101 11359049

[bb4] Bruker (1997). *SMART* and *SAINT* Bruker AXS Inc., Madison, Wisconsin, USA.

[bb5] Hagan, O. D. (2008). *Chem. Soc. Rev.* **37**, 308–319.

[bb6] Sheldrick, G. M. (1996). *SADABS* University of Göttingen, Germany.

[bb7] Sheldrick, G. M. (2008). *Acta Cryst.* A**64**, 112–122. 10.1107/S0108767307043930 18156677

[bb8] Zeng, Z. & Shreeve, J. M. (2009). *J. Fluorine Chem.* **130**, 727–732.

